# Measuring Coupling of Rhythmical Time Series Using Cross Sample Entropy and Cross Recurrence Quantification Analysis

**DOI:** 10.1155/2017/7960467

**Published:** 2017-10-22

**Authors:** John McCamley, William Denton, Elizabeth Lyden, Jennifer M. Yentes

**Affiliations:** ^1^MORE Foundation, 18444 N. 25th Ave, Suite 110, Phoenix, AZ 85023, USA; ^2^Center for Research in Human Movement Variability, University of Nebraska Omaha, 6160 University Drive, Omaha, NE 68182-0860, USA; ^3^College of Public Health, University of Nebraska Medical Center, 984355 Medical Center, Omaha, NE 68198-4355, USA

## Abstract

The aim of this investigation was to compare and contrast the use of cross sample entropy (xSE) and cross recurrence quantification analysis (cRQA) measures for the assessment of coupling of rhythmical patterns. Measures were assessed using simulated signals with regular, chaotic, and random fluctuations in frequency, amplitude, and a combination of both. Biological data were studied as models of normal and abnormal locomotor-respiratory coupling. Nine signal types were generated for seven frequency ratios. Fifteen patients with COPD (abnormal coupling) and twenty-one healthy controls (normal coupling) walked on a treadmill at three speeds while breathing and walking were recorded. xSE and the cRQA measures of percent determinism, maximum line, mean line, and entropy were quantified for both the simulated and experimental data. In the simulated data, xSE, percent determinism, and entropy were influenced by the frequency manipulation. The 1 : 1 frequency ratio was different than other frequency ratios for almost all measures and/or manipulations. The patients with COPD used a 2 : 3 ratio more often and xSE, percent determinism, maximum line, mean line, and cRQA entropy were able to discriminate between the groups. Analysis of the effects of walking speed indicated that all measures were able to discriminate between speeds.

## 1. Introduction

It has long been suggested that various biological rhythms, such as walking and breathing rhythms, have some degree of synchrony. Hey et al. [1] noted in a 1966 paper that breathing frequency was often a submultiple of stepping frequency. Subsequent studies observed this synchrony of breathing and walking or running rhythms, and it has become commonly referred to as locomotor-respiratory coupling [2–5]. The synchrony of breathing and stepping rhythms was considered to be present when the interval between heel strike and the beginning of breath inspiration or expiration was constant for a series of breaths [2]. Synchrony of breathing rhythms was observed for 8 of 15 participants while walking on a treadmill [3]. Stepping frequency influenced breathing frequency even when no rhythmical coupling existed [4]. These findings and those of many others [5–7] have soundly established that locomotor-respiratory coupling exists during walking in humans.

Previously, locomotor-respiratory coupling has been investigated using standard deviation of the interval between inspiration and heel strike [2], the percentage of inspirations starting at the same stage of the walking cycle [8], creating a cross-correlogram of breathing period versus step period [3], and discrete relative phase and return maps [6]. These tools, while providing useful information concerning the relationships between measured breathing and walking rhythms, may not be appropriate for biological systems that are nonstationary and noisy [9]. It has been suggested that biological rhythms may be considered nonlinear in nature [10]. It is appropriate, therefore, that the coupling between biological rhythms be investigated using measures that can assess the nonlinear nature of the rhythms under observation. Two such measures are cross sample entropy [11] and cross recurrence quantification analysis [12].

The concept of entropy as a means to describe the randomness of a finite sequence was described by Kolmogorov and Uspenskii [13]. Pincus [14] introduced approximate entropy to quantify regularity using relatively few data points. Pincus and Singer [15] developed cross approximate entropy as an extended form of approximate entropy to quantify asynchrony or conditional irregularity in interconnected networks [16]. Sample entropy was subsequently introduced to overcome a bias in approximate entropy caused by the counting of self matches [11]. It was noted that cross approximate entropy, while not affected by self matches, lacked relative consistency [11]. In a similar manner to cross approximate entropy, cross sample entropy (xSE) was developed from sample entropy to allow the measurement of asynchrony with relative consistency. xSE provides a method of determining if patterns that are similar within one data series are also similar in another data series [11]. It has been used to examine relationships in situations as diverse as stock markets [17], voice disorders [18], and renal sympathetic nerve activity in rats [19].

The use of recurrence plots to analyze experimental time series was originally proposed by Eckmann et al. [20]. A recurrence plot is a tool that reduces a potentially high-dimensional, nonlinear, dynamical system into a two-dimensional representation of points, revealing recurring patterns or trajectories within the system. While a visual inspection of such plots exposes many interesting qualitative features, further quantitative analysis was proposed by Zbilut and Webber [21]. For example, quantifying the recurrence rate of these plots will measure how often a system revisits a state it already visited. Cross recurrence quantification analysis (cRQA) is an adaptation of recurrence quantification. Cross recurrences between two signals are found by calculating the distances between all points in one series with all points of another series, rather than within one system to itself [12, 22]. The cRQA plot provides a visual representation of the coupling of two different time series on one time scale. This visualization and the measures which are subsequently extracted do not provide a direct measure of the strength of coupling between two signals. When the cRQA measures are viewed in combination they do give useful information concerning how the two signals relate to one another over time, from which information about coupling may be deduced. cRQA has previously been used to investigate interpersonal coordination [22], to identify cover songs [23], and for intra- and interpersonal interlimb coordination [24].

The purpose of this paper was to compare and contrast the use of xSE and cRQA measures, for the coupling assessment of rhythmical patterns. The measures were first assessed using simulated signals with three known fluctuations in frequency, amplitude, and a combination of both. Further, locomotor-respiratory coupling for two experimental groups was studied as models of normal and abnormal coupling. The same measures were used to assess the coupling of breathing and walking rhythms for older healthy subjects and patients with chronic obstructive pulmonary disease (COPD). COPD affects the breathing rhythm [25] and walking rhythms [26] of patients. However, it is not well understood whether the observed changes in walking rhythms are coupled to the altered breathing rhythms of patients with COPD.

## 2. Methods

### 2.1. Simulated Data Analysis

Fluctuations in biological signals may occur in a variety of ways. For example, changes in walking speed may be achieved through altered frequency (e.g., step time), amplitude (e.g., step length), or a combination of both. The frequency of the signal (e.g., step time) may change over time, such as the continual variations that may occur in stride frequency. An alternative way to change walking speed is to change the amplitude, the step length. In reality, both will generally change. It is important to understand the effect that alterations in amplitude, frequency, or both have on the ability of analysis methods to determine the nature of coupling between signals. To understand the ability of different measures to differentiate between signals, it is necessary to assess the differences in measures calculated from simulated signals with known levels of complexity.

**Figure 1:**
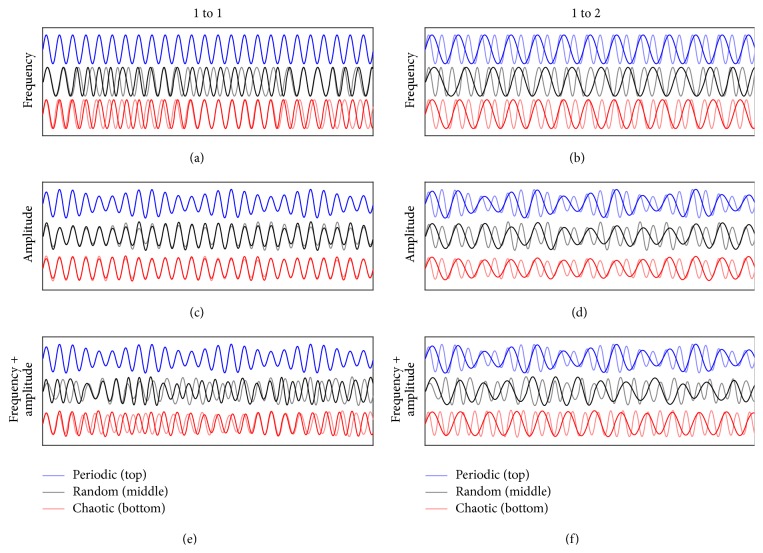
Simulated sinusoidal signals used for analysis, which consist of periodic (top), chaotic (middle), and random (bottom) varying frequency (a, b), amplitudes (c, d), or both (e, f). Two of the seven different frequency ratios used in the analysis are represented: 1 : 1 (a, c, e) and 1 : 2 (b, d, f), where the lower frequency signal is denoted by a solid, dark line, and the higher frequency signal (1 Hz) is denoted by a solid, lighter line. The signals with a varying frequency all fluctuated between a minimum of 0.83*∗f*_ratio_ and a maximum of 1.23*∗f*_ratio_ seconds (a, b), whereas the signals with varying amplitude all fluctuated between a minimum of 0.5 and a maximum of 1 unit (c, d). Additionally, the signals with both varying frequency and amplitude shared these same characteristics (e, f).

Nine different types of synthetic oscillating, quasi-sinusoidal signals were generated using custom Matlab codes (MathWorks, Inc., Natick, MA), forming a 3 × 3 matrix. The first dimension of the matrix represented the fluctuations present in the signal (periodic, chaotic, or random) and the second dimension represented the parameter(s) which varied in the signal (frequency, amplitude, or both). For each of these nine signal types, different frequency ratios (*f*_ratio_ = 1 : 1, 2 : 3, 1 : 2, 2 : 5, 1 : 3, 2 : 7, and 1 : 4) were generated. Ten signals of each ratio were generated for random and chaotic fluctuations. As the periodic signals do not vary, one signal of each ratio was generated for analysis (Figure 1). The signal with the higher frequency was constructed to have a similar frequency as walking, which for the biological data recorded averaged approximately 0.75 Hz. Thus, for each of the “5-minute” long trials at 30 Hz (9000 data points), 225 cycles would occur. The lower frequency signals varied in the number of cycles based on the frequency ratio. For example, a 2 : 3 ratio would have 225 cycles for one signal and 150 cycles for the second and a 1 : 2 ratio would have 225 cycles for one signal and 112.5 cycles for the second.

**Figure 2:**
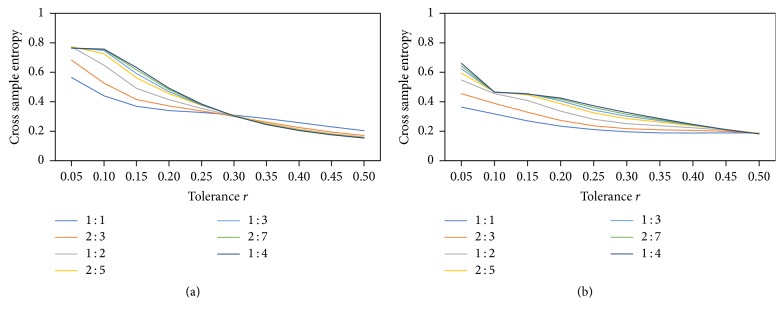
Plots of xSE for generated chaotically fluctuating signals for (a) *m* = 2 and (b) *m* = 3 for each tolerance *r* between 0.05 and 0.5, for each frequency ratio.

xSE is defined as a function of *N*, *m*, and *r*, where *N* is the data length, *m* is the vector length, and *r* is the tolerance. xSE was calculated for each signal ratio pair using custom Matlab codes (see Supplementary Data available online at https://doi.org/10.1155/2017/7960467). The procedure whereby xSE is calculated is described in detail elsewhere [11]. Values of *m* = 2 and 3 and *r* = 0.05–0.50 in increments of 0.05 were initially investigated for this analysis. Analysis of xSE values across conditions showed that, for *m* = 2 and all manipulation types (frequency, amplitude, and frequency and amplitude), there was an absence of relative consistency between signal fluctuation types (periodic, chaotic, and random) for increasing values of tolerance, *r* (Figure 2(a)). This is consistent with previous investigations of both theoretical and biological data [27]. When a value of *m* = 3 was used, consistent results were observed for all manipulations for values of *r* between 0.20 and 0.30 (Figure 2(b)). Values of *m* = 3 and *r* = 0.25 were used for all further comparisons.

To allow data series of different magnitudes to be compared they were first normalized to have a standard deviation equal to one.

cRQA analysis was performed using Matlab [28, 29] (see Supplementary Data). To generate a recurrence plot, both time series were embedded in a common phase space [30]. To find the embedded trajectory required for cRQA, time lags were determined for each time series pair, using the average mutual information algorithm [31]. The average of the time lags was used for the analysis. Embedding dimension was determined for each data series using the false nearest neighbor method [32], and the maximum value used for determining the embedded trajectory.

The Euclidean distances between all points were calculated. For each pair of time series, a radius, *r*, was calculated to provide a 2.5% recurrence. A percent recurrence (% REC) of 2.5 was selected based on previous analysis to provide a sparse recurrence plot. It has been determined that a sparse recurrence plot with low percent recurrence values provides the most information [33]. This approach eliminated % REC as a dependent variable. It was used to remove the effect different levels of recurrence may have on the other cRQA measures. A dot (*x*_*i*_, *y*_*i*_) was plotted on an *N* × *N* array, where *x*_*i*_ was a point in one time series and *y*_*i*_ was a point in the second time series which was less than *r* distance. A line on the recurrence plot was defined if two or more points were adjacent to one another. Diagonally oriented lines represent segments from both time series that run parallel for some time [34]. The following variables were defined to quantify various features of the cross recurrence plots:

- *Percent determinism* (% DET): the proportion of recurrent points that fall on diagonal lines of 2 points in length. This measure shows that for each time the two signals occur in the same region of phase space, the proportion of times they remain in the same phase space for at least one time interval. % DET represents the predictability of coupling between the two signals. A perfectly coupled system would provide a % DET value close to 100%, whereas two uncoupled signals would have a much lower value.
- *Max line*: the length of the longest diagonal line of recurrent points. A longer max line is an indication of the attractor strength [35] and shows the longest period during which the two systems occupy the same region of reconstructed phase space.
- *Mean line*: the average length of diagonal recurrence lines. A longer average line length will indicate the two signals spent on average more time in the same region of reconstructed phase space and may be an indication of stronger coupling. While max line shows the longest period during which the signals are coupled within the sample, mean line will provide an indication of the average period during which the signals are coupled.
- *cRQA entropy*: the Shannon information entropy [36] based on the distribution of the diagonal line lengths. cRQA entropy defines the probability that the length of a line will be repeated in the recurrence plot. Lower values indicated greater probability (highly repeatable) and higher entropy values indicate less probability (greater irregularity). This measure can be interpreted as the variety of patterns in which the two systems are coupled or the nature of the paths that the two systems are visiting in state space.

### 2.2. Biological Data Collection and Analysis

Fifteen patients with COPD (males = 7; 63.8 (8.0) yrs; 1.68 (0.10) m; 90.5 (32.5) kg) and 21 healthy controls (males = 6; 60.2 (6.8) yrs; 1.63 (.07) m; 74.8 (16.1) kg) were recruited for the study. Patients with COPD were recruited from the Pulmonary Studies Unit at the University of Nebraska Medical Center and the general population. COPD was determined based on reported previous diagnosis of the disease and confirmed with spirometry testing ratio of forced expiratory volume in one second to forced vital capacity (FEV1/FVC) of less than 0.7 [37]. Subjects were excluded from the study if they reported a history of musculoskeletal, cardiovascular, or neurological disease or impairment which affected their walking ability. Subjects without COPD were excluded if they reported any heart conditions. All subjects were enrolled and consented for the study under Institutional Review Board approved procedures.

Subjects wore a tight-fitting suit for data collection. Reflective spherical markers were attached to the body and suit over specific anatomic locations according to a modified Helen Hayes marker set [34]. Marker trajectories were recorded at 120 Hz using a 12-camera motion capture system (Motion Analysis Corp., Santa Rosa, CA). The anterior posterior trajectory of the marker attached to the right heel was used for further analysis in this study. Breathing data were recorded using a pulmonary testing device (K4B2, Cosmed Srl, Rome, Italy) which recorded inhalation and exhalation at 25 Hz.

Subjects' self-selected treadmill walking speed (SSWS) was determined. Following a period of at least five-minute rest and after returning to resting heart rate, subjects returned to the treadmill and completed a five-minute period of walking at SSWS and then two randomly ordered five-minute periods of walking at either ±20% of their SSWS.

All data (marker and breathing) were downsampled to 30 Hz. A custom Matlab code determined how many right heel strikes occurred within each breath cycle and reported the percentage of each frequency ratio used during each trial (see Supplementary Data). For each data series pair (breathing and marker data), xSE and cRQA measures were calculated after the data were normalized to a mean of zero and a standard deviation of one. xSE was calculated for the biological data using the same Matlab code and range of *m* and *r* values as were used with the simulated data. Inspection of xSE values for the biological data showed consistency over all speeds for both *m* = 2 and *m* = 3 when the tolerance (*r*) was greater than 0.2 times the standard deviation of the signal. It was determined that the same *m* and *r* values as were used with the simulated data were suitable for use with the biological data.

### 2.3. Statistical Analysis

Statistical analysis was performed to determine the interaction of signal fluctuation type (periodic, chaotic, or random), manipulation (frequency, amplitude, or both), and frequency ratio (1 : 1, 2 : 3, 1 : 2, 2 : 5, 1 : 3, 2 : 7, and 1 : 4) for each of the dependent variables (xSE, % DET, max line, mean line, and cRQA entropy). Linear mixed effect models were used to compare the three factors and assess interactions. The frequency ratio was considered as a repeated measure. If the 3-way interaction term was statistically significant, the analysis was stratified by manipulation, and the interaction of signal fluctuation type and frequency ratio was evaluated. If the 2-way interaction was significant, the effect of frequency ratio within type and manipulation was determined. The *p* values for pairwise comparisons of frequency ratio were adjusted using Tukey's method. A *p* value < 0.05 was considered statistically significant.

For the biological data, the dependent variables were the same as simulated data. Linear mixed effect models were used to compare the groups (patients with COPD and healthy controls) and assess interaction. Pairwise comparisons were performed between walking speeds within group and between groups within walking speeds. The *p* values for pairwise comparisons of walking speed and group were adjusted using Tukey's method. The significance level was set at *p* < 0.05.

All statistics were performed using SAS (SAS Institute, Inc., Cary, NC).

## 3. Results

### 3.1. Simulated Data

**Figure 3:**
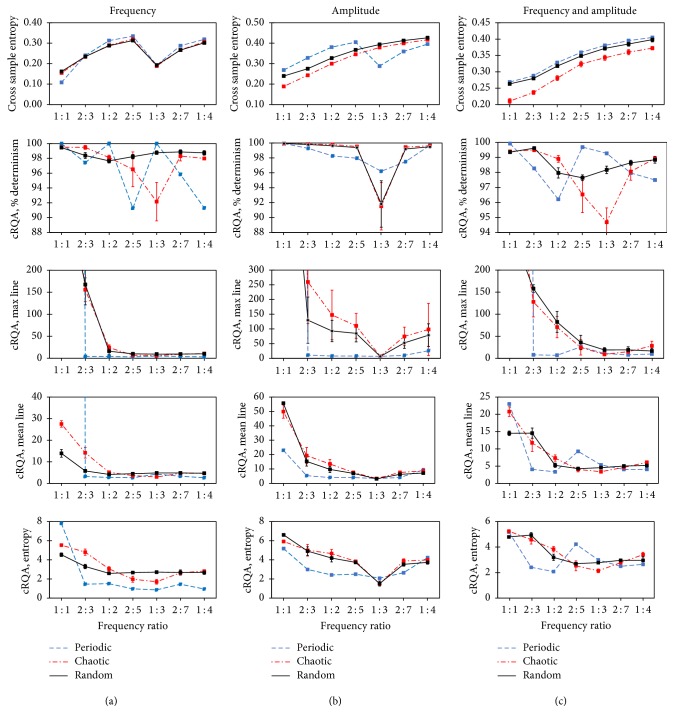
Plots of xSE and cRQA measures for simulated signals exhibiting different types of fluctuating patterns. Frequency manipulations are shown in (a), amplitude manipulations are shown in (b), and the combination of frequency and amplitude manipulations is shown in (c). Fluctuations within the signal are periodic (blue, dashed), random (black, solid), and chaotic (red, dot dash).

For xSE there was a statistically significant 3-factor interaction (*p* < 0.0001) between signal fluctuation type (periodic, chaotic, and random), manipulation (frequency, amplitude, frequency, and amplitude), and frequency ratio (1 : 1, 2 : 3, 1 : 2, 2 : 5, 1 : 3, 2 : 7, and 1 : 4). There was a statistically significant interaction between the factors, signal fluctuation type, and frequency ratio, for each level of manipulation (frequency: *p* < 0.0001; amplitude: *p* < 0.0001; and frequency/amplitude: *p* < 0.0001) (Figure 3). Pairwise comparisons of fluctuation ratio revealed significant statistical differences for both chaotic and random signal fluctuation types, for all manipulations (Supplemental Table S1).

The dependent variables from the cRQA analysis also revealed significant results; % DET, max line, mean line, and cRQA entropy all demonstrated a significant 3-factor interaction (*p* < 0.0001 for all measures), similar to xSE (Figure 3). A significant interaction between signal fluctuation type and frequency ratio was also found for each of these dependent variables (*p* < 0.0001, for all). Pairwise comparisons of fluctuation ratio revealed numerous statistical differences for each cRQA variable (Supplemental Tables S2–5).

### 3.2. Biological Data

**Figure 4:**
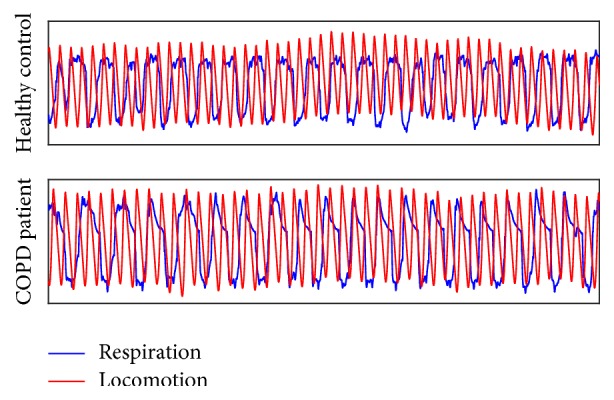
Representative data from breathing and walking from a patient with COPD and a healthy control. The red line represents the heel marker and the blue line represents flow data from breathing. Data shown are from the first minute of walking. Both time series have been normalized to a mean of zero and standard deviation of one.

The SSWS for the patients with COPD was 0.64 (0.27) m/s and 1.00 (0.22) m/s for the healthy controls (*p* < 0.001). While walking at SSWS patients with COPD had a stride frequency of 0.70 Hz and a respiration of frequency of 0.39 Hz. Healthy controls had a stride frequency of 0.87 Hz and a respiration of frequency of 0.32 Hz at SSWS. While walking at −20% SSWS patients with COPD and healthy controls had stride frequencies of 0.59 Hz and 0.72 Hz, respectively, and respiration frequencies of 0.37 Hz and 0.32 Hz respectively. When walking at +20% SWSS patients with COPD and healthy controls had stride frequencies of 0.71 Hz and 0.91 Hz, respectively, and respiration frequencies of 0.39 Hz and 0.33 Hz, respectively. The most common frequency ratio for the healthy controls was 1 : 2 at all three speeds. Healthy controls utilized a range of frequency ratios alternating between 1 : 1, 2 : 3, 1 : 2, 2 : 5, 1 : 3, and 1 : 4 (breath : strides). The most common frequency ratio for the patients with COPD was 2 : 3 at SSWS and +20% and 1 : 1 at −20%. Patients with COPD utilized frequency ratios ranging from 1 : 1, 2 : 3, 1 : 2, 2 : 5, and 1 : 3 (Figure 4).

**Figure 5:**
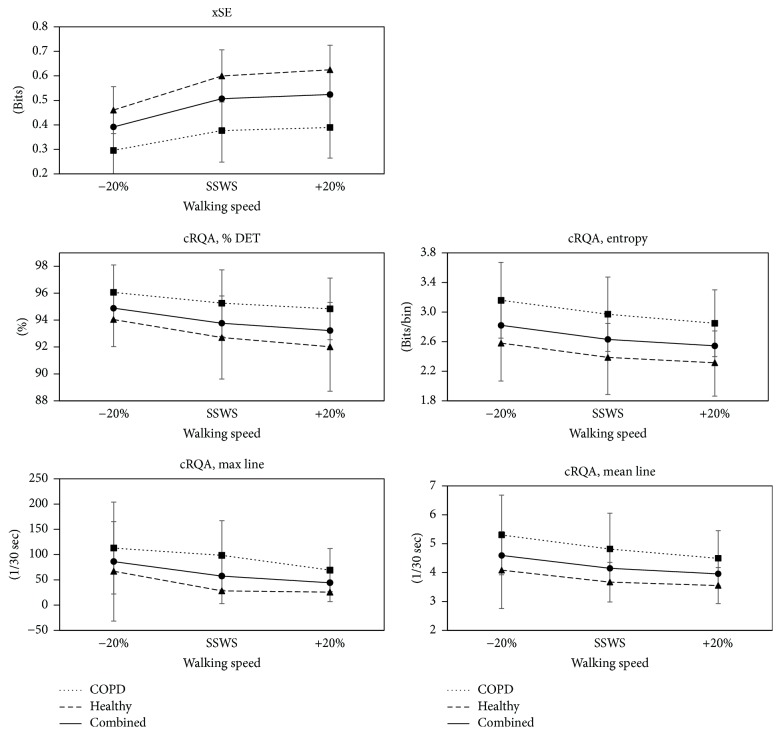
Group means for xSE and cRQA measures for patients with COPD (square with dotted line) and healthy controls (triangle with dashed line) and COPD and healthy combined (circle with solid line), at all three speeds.* Note*. SWSS is self-selected walking speed.

For xSE there was a statistically significant interaction between group (COPD, healthy controls) and walking speed (*p* = 0.004). The difference in mean xSE between groups differed based on condition. xSE was significantly reduced in patients with COPD when compared to the healthy controls (adjusted *p* < 0.001; Figure 5). When both groups were combined (effect of speed), xSE increased with speed. There was a significant difference between xSE at −20% and +20% SSWS (adjusted *p* < 0.001). Among the controls, there was* no* statistically significant difference in the mean xSE measurement between conditions −20% and +20% SSWS (adjusted *p* = 0.39).

cRQA measures were found to have main effects of group and speed (Figure 5). Patients with COPD had an increased % DET (*p* = 0.0036), a longer max line (*p* = 0.0009), a longer mean line (*p* = 0.001), and a greater cRQA entropy (*p* = 0.004) as compared to controls. When both groups are combined, % DET (*p* = 0.001), max line (*p* = 0.01), mean line (*p* < 0.0001), and cRQA entropy (*p* = 0.004) were found to have an effect of speed. Differences were observed between the slowest and fastest speeds (−20% and +20% SSWS) for % DET (*p* = 0.0001), max line (*p* = 0.009), mean line (*p* < 0.0001), and cRQA entropy (*p* = 0.0003). Differences were also noted between the slowest (−20%) and SSWS for % DET (*p* = 0.01), mean line (*p* = 0.006), and cRQA entropy (*p* = 0.02). No significant interactions were found.

## 4. Discussion

The purpose of this paper was to compare and contrast the use of xSE and cRQA measures using both simulated and experimental data. Both xSE and cRQA measures were assessed using simulated signals with regular, chaotic, and random manipulations of frequency, amplitude, and a combination of both frequency and amplitude at a range of frequency ratios. Moreover, locomotor-respiratory coupling in biological data, obtained from healthy subjects and patients with COPD, was studied as models of normal and abnormal coupling. The results of the investigation of simulated data demonstrated that while xSE was significantly different for all manipulations and all frequency ratios, cRQA measures were influenced by the type of manipulation over all ratios except 1 : 1. The measures of xSE, % DET, max line, mean line, and cRQA entropy were all able to discriminate between the biological signals of the experimental groups (normal and abnormal coupling) and speeds.

Based on the simulation results, the use of xSE on signals with both frequency and amplitude fluctuations distinguished between signal types irrespective of ratio. cRQA, max line, mean line, and entropy were able to distinguish all other ratios from 1 : 1, no matter the manipulation of the signals. The 1 : 1 frequency ratio was significantly different than all other frequency ratios for all measures and/or manipulations except for random fluctuations in signal frequency. Mean line and cRQA entropy were the cRQA measures best able to distinguish between the full range of frequency ratios for chaotic and random signals that were subjected to amplitude manipulation and chaotic signals that were subjected to combined frequency and amplitude manipulation. cRQA entropy was also able to distinguish between most frequency ratios when the signal frequencies fluctuated chaotically. The cRQA measures all showed the ability to distinguish between other frequency ratios to varying levels with cRQA entropy particularly effective for chaotically fluctuating signals and signals with randomly fluctuating amplitude.

The patients with COPD mainly utilized the 1 : 1 frequency ratio and based on the simulated and biological results, it appears that the cRQA measures of max line, mean line, and entropy are better suited to discriminate between groups and signal complexity than % DET. For these data, the values of % DET were close to 100% in many cases. This could be due to the selection of very high level of % REC, which may have limited the sensitivity of this measure especially for the quasiperiodic generated data. For this study two points were used to define a diagonal line. Further investigation of % DET for a range of % REC values and requiring diagonal lines of longer than two points may provide further insight into the utility of this measure to discriminate between these signals.

The results from the simulated data show that xSE has an apparent advantage for discriminating between signals of different levels of complexity over a range of coupling frequencies especially if a ratio of 1 : 1 is included. This may be due to the compromises that must be made to place signals of different frequencies into the same reconstructed phase space for determination of the recurrence and associated measures when calculating cRQA. For cRQA, signals were unfolded to the maximum embedding dimension [32] to ensure that all of the dynamics of the system were observed. Because it was required to explore the coupling of the signals over time it was necessary to compare the same points in time for each signal. This means that one signal will make more orbits of the attractor in the same period compared to another signal, when the coupling ratio is other than 1 : 1. In this situation, it is necessary to make a compromise when selecting an appropriate lag for construction of an attractor for both time series. In this case, the compromise was to select the lag of the combined attractor as the mean of the values determined for each time series from the minimum average mutual information. No matter what lag is used, a higher frequency signal will still orbit the attractor in fewer data points than a lower frequency signal. Using different sampling rates for each signal may solve this problem if the frequencies of each signal remain exactly constant, but as soon as the frequency of one or other signals changes, the sampling rate for that signal will no longer be appropriate. The effect of having the reconstructed attractors for signals with different frequencies embedded in a common space is apparent by the different associations between periodic, chaotic, and random signals which were observed at the 1 : 1 coupling ratio compared to those at other coupling ratios.

This would suggest that, for the analysis of the relationship between subjects' walking and breathing rhythms, it appears necessary to first have an understanding of the ratio of the signal frequencies that are present, in order to ascertain the most appropriate measure to use. A further consideration is that when walking speed changes, frequency of both stepping and breathing does not necessarily change concurrently. For example, when walking speed changes, breathing rate may not change and thus the ratio between walking and breathing will change. As subjects walk more slowly their step frequency will reduce with a tendency for breath to stride ratios closer to 1 : 1. Patients with COPD utilized a ratio of 2 : 3 most often at their self-selected and faster walking speeds and 1 : 1 at the slower speed, concurrently adapting their relative frequencies at each speed. Healthy controls in general walked faster and utilized a wider range of frequency ratios ranging over 1 : 1, 2 : 3, 1 : 2, 2 : 5, 1 : 3, and 1 : 4. As patients with COPD alter their walking speed, they have diminished capacity to independently alter their breathing rhythm in response. Another consideration is that even if the relative frequencies do not alter, the comparative patterns within the coupling can change, something that will become apparent using the appropriate analysis. Treadmill walking may have an effect on the complexity of walking patterns [38] such that a similar analysis of breathing and walking in an overground situation may show differences that cannot be detected using these methods while walking on a treadmill. Patients with COPD were heavier and walked more slowly than healthy controls. As patients with COPD are generally heavier than healthy age matched subjects a mismatch in weight was necessary to obtain a large enough sample size. An increase in sample size may allow more close matching of groups; however subjects walked at their self-selected walking speed and proportionally slower and faster speeds, to minimize the effects on the coupling of breathing and walking patterns.

xSE, % DET, max line, mean line, and cRQA entropy were able to detect changes in frequency ratios for the biological data. Patients with COPD demonstrated greater synchrony of coupling (xSE) and a longer period of time that breathing and walking were coupled (% DET, max line, and mean line). This is understandable considering their repeated use of lower frequency ratios like 1 : 1 and 2 : 3. If two signals have the same frequency, it is much more likely they will be able to remain in the same region of reconstructed phase space for a longer period than two signals of different frequencies. In addition, patients with COPD demonstrated a greater irregularity in the variety of patterns in which the two systems were coupled (cRQA entropy) at self-selected and faster walking speeds. This means that while patients with COPD had increased levels of overall coupling (% DET), the coupling occurred in a more irregular manner when these subjects were asked to walk at self-selected and a faster speed.

While this study has attempted to comprehensively investigate the effect different signal types have on the determination of coupling using xSE and cRQA, it is by no means exhaustive. Specific signal types were selected for the initial analysis. Three types of signal manipulation were chosen to represent strongly coupled (periodic), uncoupled (random), and intermediately coupled (chaotic) fluctuations. The use of further generated signals with known levels of coupling will provide a more comprehensive understanding of the utility of xSE and cRQA measures to assess strength of coupling. While xSE has previously been used to assess continuous signals [18], sample entropy is generally calculated from discrete measurements such as heart beat intervals [39] or nonperiodic data such as postural sway [40, 41]. It has been suggested that the calculation of sample entropy when used with continuous data includes a delay parameter to downsample these data [42]. Considering the application of sample entropy, the use of continuous signals may not be most appropriate for calculating xSE. While it would not be possible to compare directly stride intervals and breathing intervals of different frequencies if the coupling ratio is known, then perhaps downsampling the higher frequency intervals may be an alternative. The generated signals were obtained by manipulating a sine wave. While movement patterns are generally considered to be rhythmical they do not follow a sinusoidal pattern. The parameters *r* and *m* were selected from a range generally considered appropriate for biological data (Supplemental Data). A more robust assessment of the ability of xSE to discriminate signals would be obtained by using an increased range of values for *r* and *m*.

Similarly, determining the effect different embedding dimensions and time lags have on the cRQA measures will provide further knowledge of how these parameters will affect cRQA outcomes. In this study, the radius was adjusted to ensure the same percent recurrence on every recurrence plot. This was done as percent recurrence can have an effect on the other cRQA measures. Since percent recurrence was not different between the groups, this excludes the measure having a confounding effect on the other cRQA measures; however it prevents an understanding of the level of stochastic noise within the systems [43].

It is apparent from an inspection of the simulated data results that the frequency ratio of the signals under examination may influence these measures. The use of such measures will first require an understanding of the ratios present and the level of fluctuation of these ratios. A more appropriate method of comparing signals of differing frequencies using recurrence analysis may be to unfold each signal into its own state space. This will overcome the compromise necessary when selecting a time lag and embedding dimension for each signal. Joint recurrence quantification analysis [44] provides a method of assessing the relationship of signals when each is embedded in its own phase space. Further analysis is required to determine if the use of joint recurrence analysis will provide information useful in differentiating between signals of differing complexity that is not readily available using cRQA. This study, while it does not encompass all possible methods to assess the strength of coupling between two signals, does provide useful information from which to assess the strengths and weaknesses of two methods that are commonly thought appropriate. It is apparent that before deciding to use either method, it is necessary to have a basic understanding of the type of signals that are to be analyzed. Signals that are of differing frequencies present a unique challenge and the data may need appropriate treatment prior to analysis. Notwithstanding the limitations, the methods described in this study are both useful to describe the coupling of the biological signals presented.

The signals chosen for this study are ones that were known to occur at different frequencies and different strengths. Many other coupled biological signals also exist within the human body, and it might be expected that they can also be assessed using these methods; however further research is required to determine if this is the case. The results presented in this paper show that, with an appropriate understanding of the biological signals being assessed and the frequencies that are present, the measures of xSE and cRQA provide much useful information concerning the level and patterns of coupling which are occurring between said signals.

## Supplementary Material

Pairwise comparisons of xSE and cRQA measures for the simulated data and Matlab script used to calculate xSE.
