# Supplementary material for: Measuring Coupling of Rhythmical Time Series Using Cross Sample Entropy and Cross Recurrence Quantification Analysis
Source: Comput Math Methods Med. 2017 Oct 22;2017:7960467. doi: 10.1155/2017/7960467 (PMC5671691; doi:10.1155/2017/7960467)
Supplement: Supplementary file 1 — Pairwise comparisons of xSE and cRQA measures for the simulated data and Matlab script used to calculate xSE. [file 7960467.f1.pdf]

Supplementary Data

Pairwise comparisons of xSE and cRQA measures for the simulated data (Tables S1-5).

Note: A red box indicate manipulations that significantly affected the ratio of 1:1 to other ratios. A blue box indicates manipulations that significantly affected the ratio of 1:2 to other ratios. A yellow box indicates manipulations that significantly affected the ratio of 1:3 to other ratios. A green box indicates manipulations that had an effect on the majority of ratio comparisons.

Table S1. Adjusted p-values for the pairwise comparisons of fluctuation ratio within each level of manipulation and signal fluctuation type for the simulated data for cRQA radius. Table excludes the periodic signal fluctuation type. Significantly different ($p<0.05$) comparisons are shown in bold print. Note: M = manipulation and T = signal fluctuation type.

Comparison	M: Frequency<br>T: Chaotic	M: Frequency<br>T: Random	M: Amplitude<br>T: Chaotic	M: Amplitude<br>T: Random	M: Freq/Amp<br>T: Chaotic	M: Freq/Amp<br>T: Random
1:1 to 1:2	**<.0001**	0.14	**<.0001**	**<.0001**	**<.0001**	**<.0001**
1:1 to 1:3	**<.0001**	1.0	**<.0001**	**<.0001**	**<.0001**	**<.0001**
1:1 to 1:4	**<.0001**	**<.0001**	**<.0001**	**<.0001**	**<.0001**	**<.0001**
1:1 to 2:3	**<.0001**	1.0	**<.0001**	**<.0001**	**<.0001**	**<.0001**
1:1 to 2:5	**<.0001**	0.27	**<.0001**	**<.0001**	**<.0001**	**<.0001**
1:1 to 2:7	**<.0001**	0.19	**<.0001**	**<.0001**	**<.0001**	**<.0001**
1:2 to 1:3	**<.0001**	0.07	**<.0001**	**<.0001**	**<.0001**	1.0
1:2 to 1:4	0.11	**<.0001**	**0.04**	1.0	0.86	1.0
1:2 to 2:3	0.63	0.11	**0.01**	**0.001**	**<.0001**	**0.004**
1:2 to 2:5	**0.03**	1.0	1.0	1.0	**<.0001**	1.0
1:2 to 2:7	**0.04**	**<.0001**	1.0	1.0	**0.04**	0.38
1:3 to 1:4	0.06	**<.0001**	**<.0001**	**<.0001**	**0.0004**	0.90
1:3 to 2:3	**0.003**	1.0	**<.0001**	**<.0001**	0.07	**0.01**
1:3 to 2:5	0.20	0.14	**<.0001**	**<.0001**	1.0	1.0
1:3 to 2:7	0.14	0.35	**<.0001**	**<.0001**	0.44	0.18
1:4 to 2:3	0.94	**<.0001**	**<.0001**	**0.0005**	**<.0001**	**0.0003**
1:4 to 2:5	1.0	**<.0001**	0.10	1.0	**0.0008**	0.75
1:4 to 2:7	1.0	**0.02**	**0.01**	1.0	0.12	0.84
2:3 to 2:5	0.68	0.21	**0.003**	**0.0001**	**0.04**	**0.03**
2:3 to 2:7	0.78	0.24	**0.04**	**0.0008**	**0.0002**	**<.0001**
2:5 to 2:7	1.0	**0.0003**	0.97	1.0	0.58	0.10

Table S2. Adjusted p-values for the pairwise comparisons of fluctuation ratio within each level of manipulation and signal fluctuation type for the simulated data for cRQA %DET. Table excludes the periodic signal fluctuation type. Significantly different ($p<0.05$) comparisons are shown in bold print. Note: M = manipulation and T = signal fluctuation type.

Comparison	M: Frequency<br>T: Chaotic	M: Frequency<br>T: Random	M: Amplitude<br>T: Chaotic	M: Amplitude<br>T: Random	M: Freq/Amp<br>T: Chaotic	M: Freq/Amp<br>T: Random
1:1 to 1:2	**<.0001**	0.14	**<.0001**	**<.0001**	**<.0001**	**<.0001**
1:1 to 1:3	**<.0001**	1.0	**<.0001**	**<.0001**	**<.0001**	**<.0001**
1:1 to 1:4	**<.0001**	**<.0001**	**<.0001**	**<.0001**	**<.0001**	**<.0001**
1:1 to 2:3	**<.0001**	1.0	**<.0001**	**<.0001**	**<.0001**	**<.0001**
1:1 to 2:5	**<.0001**	0.27	**<.0001**	**<.0001**	**<.0001**	**<.0001**
1:1 to 2:7	**<.0001**	0.19	**<.0001**	**<.0001**	**<.0001**	**<.0001**
1:2 to 1:3	**<.0001**	0.07	**<.0001**	**<.0001**	**<.0001**	1.0
1:2 to 1:4	0.11	**<.0001**	**0.04**	1.0	0.86	1.0
1:2 to 2:3	0.63	0.11	**0.01**	**0.001**	**<.0001**	**0.004**
1:2 to 2:5	**0.03**	1.0	1.0	1.0	**<.0001**	1.0
1:2 to 2:7	**0.04**	**<.0001**	1.0	1.0	**0.004**	0.38
1:3 to 1:4	0.06	**<.0001**	**<.0001**	**<.0001**	**0.0004**	0.90
1:3 to 2:3	**0.003**	1.0	**<.0001**	**<.0001**	0.10	0.01
1:3 to 2:5	0.20	0.14	**<.0001**	**<.0001**	1.0	1.0
1:3 to 2:7	0.14	0.35	**<.0001**	**<.0001**	0.44	0.18
1:4 to 2:3	0.94	**<.0001**	**<.0001**	**0.0005**	**<.0001**	**0.0003**
1:4 to 2:5	1.0	**<.0001**	0.10	1.0	**0.0008**	0.75
1:4 to 2:7	1.0	**0.02**	**0.009**	1.0	0.12	0.84
2:3 to 2:5	0.68	0.21	**0.003**	**0.0001**	**0.04**	**0.03**
2:3 to 2:7	0.78	0.24	**0.04**	**0.0008**	**0.0002**	**<.0001**
2:5 to 2:7	1.0	**0.0003**	1.0	1.0	0.58	0.10

Table S3. Adjusted p-values for the pairwise comparisons of fluctuation ratio within each level of manipulation and signal fluctuation type for the simulated data for cRQA Max Line. Table excludes the periodic signal fluctuation type. Significantly different ($p<0.05$) comparisons are shown in bold print. Note: M = manipulation and T = signal fluctuation type.

Comparison	M: Frequency<br>T: Chaotic	M: Frequency<br>T: Random	M: Amplitude<br>T: Chaotic	M: Amplitude<br>T: Random	M: Freq/Amp<br>T: Chaotic	M: Freq/Amp<br>T: Random
1:1 to 1:2	**<.0001**	**<.0001**	**<.0001**	**<.0001**	**<.0001**	**<.0001**
1:1 to 1:3	**<.0001**	**<.0001**	**<.0001**	**<.0001**	**<.0001**	**<.0001**
1:1 to 1:4	**<.0001**	**<.0001**	**<.0001**	**<.0001**	**<.0001**	**<.0001**
1:1 to 2:3	**<.0001**	**<.0001**	**<.0001**	**<.0001**	**<.0001**	**<.0001**
1:1 to 2:5	**<.0001**	**<.0001**	**<.0001**	**<.0001**	**<.0001**	**<.0001**
1:1 to 2:7	**<.0001**	**<.0001**	**<.0001**	**<.0001**	**<.0001**	**<.0001**
1:2 to 1:3	0.81	1.0	0.28	0.09	**<.0001**	**<.0001**
1:2 to 1:4	0.94	1.0	1.0	1.0	**0.01**	**<.0001**
1:2 to 2:3	**<.0001**	**<.0001**	0.55	0.89	**0.0002**	**<.0001**
1:2 to 2:5	0.84	1.0	1.0	1.0	**0.004**	**<.0001**
1:2 to 2:7	0.91	1.0	0.90	0.84	**0.0003**	**<.0001**
1:3 to 1:4	1.0	1.0	0.76	0.23	0.66	1.0
1:3 to 2:3	**<.0001**	**<.0001**	**0.03**	**0.003**	**<.0001**	**<.0001**
1:3 to 2:5	1.0	1.0	0.64	0.16	0.83	0.49
1:3 to 2:7	1.0	1.0	0.93	0.74	1.0	1.0
1:4 to 2:3	**<.0001**	**<.0001**	0.15	0.64	**<.0001**	**<.0001**
1:4 to 2:5	1.0	1.0	1.0	1.0	1.0	0.33
1:4 to 2:7	1.0	1.0	1.0	0.98	0.92	1.0
2:3 to 2:5	**<.0001**	**<.0001**	0.22	0.76	**<.0001**	**<.0001**
2:3 to 2:7	**<.0001**	**<.0001**	0.06	0.17	**<.0001**	**<.0001**
2:5 to 2:7	1.0	1.0	1.0	0.94	0.98	0.50

Table S4. Adjusted p-values for the pairwise comparisons of fluctuation ratio within each level of manipulation and signal fluctuation type for the simulated data for cRQA Mean Line. Table excludes the periodic signal fluctuation type. Significantly different ($p<0.05$) comparisons are shown in bold print. Note: M = manipulation and T = signal fluctuation type.

Comparison	M: Frequency<br>T: Chaotic	M: Frequency<br>T: Random	M: Amplitude<br>T: Chaotic	M: Amplitude<br>T: Random	M: Freq/Amp<br>T: Chaotic	M: Freq/Amp<br>T: Random
1:1 to 1:2	**<.0001**	**<.0001**	**<.0001**	**<.0001**	**<.0001**	**<.0001**
1:1 to 1:3	**<.0001**	**<.0001**	**<.0001**	**<.0001**	**<.0001**	**<.0001**
1:1 to 1:4	**<.0001**	**<.0001**	**<.0001**	**<.0001**	**<.0001**	**<.0001**
1:1 to 2:3	**<.0001**	**<.0001**	**<.0001**	**<.0001**	**<.0001**	**1.0000**
1:1 to 2:5	**<.0001**	**<.0001**	**<.0001**	**<.0001**	**<.0001**	**<.0001**
1:1 to 2:7	**<.0001**	**<.0001**	**<.0001**	**<.0001**	**<.0001**	**<.0001**
1:2 to 1:3	**0.002**	0.41	**<.0001**	**<.0001**	**<.0001**	0.32
1:2 to 1:4	0.97	0.67	**0.01**	**0.007**	0.25	1.0
1:2 to 2:3	**<.0001**	**0.0002**	**0.002**	**<.0001**	**<.0001**	**<.0001**
1:2 to 2:5	0.07	0.97	**0.001**	**0.003**	**<.0001**	0.05
1:2 to 2:7	0.98	0.41	**0.001**	**<.0001**	**<.0001**	0.99
1:3 to 1:4	**0.04**	1.0	**0.005**	**<.0001**	**<.0001**	0.45
1:3 to 2:3	**<.0001**	0.09	**<.0001**	**<.0001**	**<.0001**	**<.0001**
1:3 to 2:5	0.89	0.92	0.05	**<.0001**	0.87	0.97
1:3 to 2:7	**0.03**	1.0	0.05	**0.0005**	0.21	0.75
1:4 to 2:3	**<.0001**	**0.03**	**<.0001**	**<.0001**	**<.0001**	**<.0001**
1:4 to 2:5	0.42	0.99	0.99	1.0	**0.005**	0.08
1:4 to 2:7	1.0	1.0	0.99	0.84	0.11	1.0
2:3 to 2:5	**<.0001**	**0.004**	**<.0001**	**<.0001**	**<.0001**	**<.0001**
2:3 to 2:7	**<.0001**	0.09	**<.0001**	**<.0001**	**<.0001**	**<.0001**
2:5 to 2:7	0.39	0.91	1.0	0.95	0.90	0.22

Table S5. Adjusted p-values for the pairwise comparisons of fluctuation ratio within each level of manipulation and signal fluctuation type for the simulated data for cRQA Entropy. Table excludes the periodic signal fluctuation type. Significantly different ($p<0.05$) comparisons are shown in bold print. Note: M = manipulation and T = signal fluctuation type.

Comparison	M: Frequency T: Chaotic	M: Frequency T: Random	M: Amplitude T: Chaotic	M: Amplitude T: Random	M: Freq/Amp T: Chaotic	M: Freq/Amp T: Random
1:1 to 1:2	**<.0001**	**<.0001**	**<.0001**	**<.0001**	**<.0001**	**<.0001**
1:1 to 1:3	**<.0001**	**<.0001**	**<.0001**	**<.0001**	**<.0001**	**<.0001**
1:1 to 1:4	**<.0001**	**<.0001**	**<.0001**	**<.0001**	**<.0001**	**<.0001**
1:1 to 2:3	**<.0001**	**<.0001**	**<.0001**	**<.0001**	**<.0001**	0.51
1:1 to 2:5	**<.0001**	**<.0001**	**<.0001**	**<.0001**	**<.0001**	**<.0001**
1:1 to 2:7	**<.0001**	**<.0001**	**<.0001**	**<.0001**	**<.0001**	**<.0001**
1:2 to 1:3	**<.0001**	0.62	**<.0001**	**<.0001**	**<.0001**	**<.0001**
1:2 to 1:4	0.29	0.91	**0.0014**	**0.006**	**0.005**	0.06
1:2 to 2:3	**<.0001**	**<.0001**	0.29	**<.0001**	**<.0001**	**<.0001**
1:2 to 2:5	**<.0001**	0.94	**<.0001**	**0.005**	**<.0001**	**<.0001**
1:2 to 2:7	**0.004**	0.88	**<.0001**	**<.0001**	**<.0001**	0.06
1:3 to 1:4	**<.0001**	1.0	**<.0001**	**<.0001**	**<.0001**	0.26
1:3 to 2:3	**<.0001**	**<.0001**	**<.0001**	**<.0001**	**<.0001**	**<.0001**
1:3 to 2:5	0.16	1.0	**<.0001**	**<.0001**	**0.01**	0.94
1:3 to 2:7	**<.0001**	1.0	**<.0001**	**<.0001**	**<.0001**	0.26
1:4 to 2:3	**<.0001**	**<.0001**	**<.0001**	**<.0001**	**<.0001**	**<.0001**
1:4 to 2:5	**<.0001**	1.0	0.95	1.0	**<.0001**	**0.02**
1:4 to 2:7	0.63	1.0	0.98	0.55	**<.0001**	1.00
2:3 to 2:5	**<.0001**	**<.0001**	**<.0001**	**<.0001**	**<.0001**	**<.0001**
2:3 to 2:7	**<.0001**	**<.0001**	**<.0001**	**<.0001**	**<.0001**	**<.0001**
2:5 to 2:7	**<.0001**	1.0	1.0	0.58	0.29	**0.02**

Matlab script used to calculate Cross Sample Entropy (xSE)

```matlab
function xSE = xSamp_Ent(x,y,m,R,norm)
% xSE = xSamp_Ent(x,y,m,R,norm)

% Function to calculate cross sample entropy for 2 data series using the
% method described by Richman and Moorman (2000). J McCamley - Sept, 2015;

% Inputs
% x - first data series
% y - second data series
% m - vector length for matching (usually 2 or 3)
% R - R tolerance to find matches (as a proportion of the average of the
%     SDs of the data sets, usually between 0.15 and 0.25)
% norm - normalization to perform
%       1 = max rescale/unit interval (data ranges in value from 0 - 1)
%       2 = mean/Zscore (used when data is more variable or has outliers)
%       normalized data has SD = 1. This is best for cross sample entropy.
%       Set to any value other than 1 or 2 to not normalize/rescale the
%       data

% Check both sets of data are the same length
xl = length(x);
yl = length(y);
if xl ~= yl
    disp('The data series need to be the same length!')
end
N = length(x);
% normalize the data ensure data fits in the same "space"
```

```
if norm == 1 %normalize data to have a range 0 - 1
    xn = (x - min(x))/(max(x) - min(x));
    yn = (y - min(y))/(max(y) - min(y));
    r = R * ((std(xn)+std(yn))/2);
elseif norm == 2 % normalize data to have a SD = 1, and mean = 0
    xn = (x - mean(x))/std(x);
    yn = (y - mean(y))/std(y);
    r = R;
else disp('These data will not be normalized')
end

fprintf(' 00.00%%');
for i = 1:N-m
    for j = 1:N-m
        for k = 1:m+1;
            dij(k) = abs(xn(i+k-1)-yn(j+k-1));
        end
        di(j) = max(dij(1:m));
        di1(j) = max(dij(1:m+1));
    end
    d = find(di<=r); % find the vectors of length 'm' that are less than "r"
distant from one another
    d1 = find(di1<=r); % find the vectors of length 'm+1' that are less than
"r" distant from one another
    nm = length(d);
    Bm(i) = nm/(N-m);
    nm1 = length(d1);
    Am(i) = nm1/(N-m);
```

```
    if mod(i,1000) == 0
        fprintf(repmat('\b',1,7));
        fprintf('%3.0d',floor(i/(N-m)*100));
        fprintf('.%1.0f',floor(mod((i/(N-m))*100,1)*10));
        fprintf('%1.0f',floor(mod((i/(N-m))*1000,1)*10));
        fprintf('%%');
    end
end
fprintf(repmat('\b',1,7));
fprintf('100.00%%');
fprintf('\r');

Bmr = sum(Bm)/(N-m);
Amr = sum(Am)/(N-m);

xSE = -log(Amr/Bmr);
end
```

Matlab script used to calculate cross recurrence quantification analysis (cRQA)

```
function [RP, RESULTS]=RQA(DATA,TYPE,EMB,DEL,ZSCORE,NORM,SETPARA,SETVALUE)
%  [RP, RESULTS]=RQA(DATA,TYPE,EMB,DEL,ZSCORE,NORM,SETPARA,SETVALUE)
%
%  Computes a recurrence plot for either recurrence quantification analysis
%  (RQA), cross recurrence quantification analysis (cRQA), jount recurrence
%  quantification analysis (jRQA), or multidimensional recurrence
%  quantification analysis (mdRQA). Either radius or target recurrence can
%  be set.
%
%
%  Inputs:
%
%  DATA is a double-variable with each dimension of the to-be-analyzed
%  signal as a row of numbers in a separate column. If too many columns are
%  present for the TYPE of analysis selected, the other columns will be
%  ignored (i.e. for 'cRQA' only the first two columns will be used).
%
%  TYPE is a string indicating which type of RQA to run (i.e. 'RQA',
%  'cRQA', 'jRQA', 'mdRQA').
%  The default value is TYPE = 'RQA'.
%
%  EMB is the number of embedding dimensions (i.e., EMB = 1 would be no
%  embedding via time-delayed surrogates, just using the provided number of
%  colums as dimensions.
%  The default value is EMB = 1.
%
%  DEL is the delay parameter used for time-delayed embedding (if EMB > 1).
%  The default value is DEL = 1.
%
```

```
%  ZSCORE indicates, whether the data (i.e., the different columns of DATA,
%  being the different signals or dimensions of a signal) should be
%  z-scored before performing MdRQA:
%    0 - no z-scoring of DATA
%    1 - z-score columns of DATA
%  The default value is ZSCORE = 0.
%
%  NORM is the type of norm by with the phase-space is normalized. The
%  following norms are available:
%    'euc' - Euclidean distance norm
%    'max' - Maximum distance norm
%    'min' - Minimum distance norm
%    'non' - no normalization of phase-space
%  The default value is NORM = 'non'.
%
%  SETPARA is the parameter which you would like to set a target value for
%  the recurrence plot (i.e. 'radius' or 'recurrence').
%  The default value is SETPARA = 'radius'.
%
%  SETVALUE sets the value of the selected parameter. If SETVALUE = 1, then
%  the radius will be set to 1 if SETPARA = 'radius' or the radius will be
%  adjusted until the recurrence is equal to 1 if SETPARA = 'recurrence'.
%  The default value if SETPARA = 'radius' is 1.
%  The default value if SETPARA = 'recurrence' is 2.5.
%
%
% Outputs:
%
%  RP is a matrix holding the resulting recurrence plot.
```

```
%
%  RESULTS is a structure holding the following recurrence variables:
%    1.  DIM    - dimension of the input data (used for mdRQA)
%    2.  EMB    - embedding dimension used in the calculation of the
%                 distance matrix
%    3.  DEL    - time lag used in the calculation of the distance matrix
%    4.  RADIUS - radius used for the recurrence plot
%    5.  NORM   - type of normilization used for the distance matrix
%    6.  ZSCORE - whether or not zscore was used
%    7.  Size   - size of the recurrence plot
%    8.  %REC   - percentage of recurrent points
%    9.  %DET   - percentage of diagonally adjacent recurrent points
%    10. MeanL  - average length of adjacent recurrent points
%    11. MaxL   - maximum length of diagonally adjacent recurrent points
%    12. EntrL  - Shannon entropy of distribution of diagonal lines
%    13. %LAM   - percentage of vertically adjacent recurrent points
%    14. MeanV  - average length of diagonally adjacent recurrent points
%    15. MaxV   - maximum length of vertically adjacent recurrent points
%    16. EntrV  - Shannon entropy of distribution of vertical lines
%    17. EntrW  - Weighted entropy of distribution of vertical weighted sums
%
%
% Reference:
%
% Wallot, S., Roepstorff, A., & Monster, D. (2016). Multidimensional
% Recurrence Quantification Analysis (MdRQA) for the analysis of
% multidimensional time-series: A software implementation in MATLAB and its
% application to group-level data in joint action. Frontiers in Psychology,
% 7, 1835. http://dx.doi.org/10.3389/fpsyg.2016.01835
```

```
%
% Eroglu, D., Peron, T. K. D., Marwan, N., Rodrigues, F. A., Costa, L. D.
% F., Sebek, M., ... & Kurths, J. (2014). Entropy of weighted recurrence
% plots. Physical Review E, 90(4), 042919.


% Version:
%
% v1.0, 28. July 2016
% by Sebastian Wallot, Max Planck Insitute for Empirical Aesthetics,
Frankfurt, Germany
%  & Dan M?nster, Aarhus University, Aarhus, Denmark
%
% v1.1, 06. July 2017
% by Will Denton (21denton@gmail.com), Troy Rand (troyrand@gmail.com), and
%  Casey Wiens (cwiens32@gmail.com), Biomechanics Research Building,
%  University of Nebraska at Omaha. Changes include cleaning up some
%  errors, making the default input arguments function correctly,
%  incorporating other types of RQA (e.g. RQA, CRQA, JRQA), allowing %REC
%  to be set instead of radius, incorporating weighted recurrence plots,
%  and adding weighted entropy.

%% Set default parameters if no input exists
% If SETPARA is not specified, set to 'radius'
if nargin < 7 || isempty(SETPARA)
    SETPARA = 'radius';
end

```

```
% If SETVALUE is not specified, set to 1 if radius is set or 2.5 if perRec is
set
if nargin < 8 || isempty(SETVALUE)
    switch lower(SETPARA)
        case {'radius','rad', 1}
            radius = 1;
            runSetRad = 0;
        case {'perrec','recurrence', 2}
            radiusStart = 0.01;
            radiusEnd = 0.5;
            runSetRad = 1;
            SETVALUE = 2.5;
    end
else
    switch lower(SETPARA)
        case {'radius','rad', 1}
            radius = SETVALUE;
            runSetRad = 0;
        case {'perrec','recurrence', 2}
            radiusStart = 0.01;
            radiusEnd = 0.5;
            runSetRad = 1;
    end
end

% If NORM is not specified, set to 'non'
if nargin < 6 || isempty(NORM)
    NORM = 'non';
```

```
end

% If ZSCORE is not specified, set to 0
if nargin < 5 || isempty(ZSCORE)
    ZSCORE = 0;
end

% If DEL is not specified, set to 1
if nargin < 4 || isempty(DEL)
    DEL = 1;
end

% If EMB is not specified, set to 1
if nargin < 3 || isempty(EMB)
    EMB = 1;
end

% If EMB is not specified, set to 1
if nargin < 2 || isempty(TYPE)
    TYPE = 'RQA';
end

% If z score is selected then z score the data
if ZSCORE
    DATA = zscore(DATA);
end

```

```
%% Begin code
% Set DIM and select proper column(s) of data if too many exist
c = size(DATA,2);
switch upper(TYPE)
    case 'RQA'
        DIM = 1;
        if c > 1
            DATA = DATA(:,1);
            warning('More than one column of data. Only using first
column.');
        end
    case {'CRQA','CROSS'}
        DIM = 2;
        if c > 2
            DATA = DATA(:,1:2);
            warning('More than two columns of data. Only using first two
columns.');
        end
    case {'JRQA','JOINT'}
        DIM = c;
        if c < 2
            error('Input data must have at least two columns.');
        end
    case {'MDRQA','MD','MULTI'}
        DIM = c;
end

% Embed the data
```

```
if EMB > 1
    for i = 1:EMB
        tempDATA(1:length(DATA)-(EMB-1)*DEL,1+DIM*(i-1):DIM*i) = DATA(1+(i-
1)*DEL:length(DATA)-(EMB-i)*DEL,:);
    end
    DATA = tempDATA;
    clear tempDATA
end

% Calculate distance matrix based on the type of RQA
switch upper(TYPE)
    case 'RQA'
        a{1}=pdist2(DATA,DATA);
        a{1}=abs(a{1})*-1;
    case {'CRQA','CROSS'}
        a{1}=pdist2(DATA(:,1:DIM:end),DATA(:,2:DIM:end));
        a{1}=abs(a{1})*-1;
    case {'JRQA','JOINT'}
        for i = 1:c
            a{i}=pdist2(DATA(:,i:DIM:end),DATA(:,i:DIM:end));
            a{i}=abs(a{i})*-1;
        end
    case {'MDRQA','MD','MULTI'}
        a{1}=pdist2(DATA,DATA);
        a{1}=abs(a{1})*-1;
end

% Normalize distance matrix
```

```
if contains(NORM, 'euc')
    for i = 1:length(a)
        b = mean(a{i}(a{i}<0));
        b = -sqrt(abs(((b^2)+2*(DIM*EMB))));
        a{i} = a{i}/abs(b);
    end
elseif contains(NORM, 'min')
    for i = 1:length(a)
        b = max(a{i}(a{i}<0));
        a{i} = a{i}/abs(b);
    end
elseif contains(NORM, 'max')
    for i = 1:length(a)
        b = min(a{i}(a{i}<0));
        a{i} = a{i}/abs(b);
    end
elseif contains(NORM, 'non')
    % do nothing
else
    error('No appropriate norm parameter specified.');
end

% Create weighted recurrence plot
wrp = a;
for i = 1:size(a,2)-1
    wrp{i+1} = wrp{i}.*wrp{i+1};
end
if i
```

```
    wrp = -(abs(wrp{i+1})).^(1/(i+1));
end
if iscell(wrp)
    wrp = wrp{1};
end

% Calculate recurrence plot
switch lower(SETPARA)
    case {'radius','rad', 1}
        [perRec, diag_hist, vertical_hist,A] = recurrenceMethod(a,radius);
    case {'perrec','recurrence', 2}
        [perRec, diag_hist, vertical_hist, radius, A] =
setRadius(radiusStart);
end

%% Calculate RQA variabes
RESULTS.DIM = DIM;
RESULTS.EMB = EMB;
RESULTS.DEL = DEL;
RESULTS.RADIUS = radius;
RESULTS.NORM = NORM;
RESULTS.ZSCORE = ZSCORE;
RESULTS.Size=length(A);
RESULTS.REC = perRec;
if RESULTS.REC > 0
    RESULTS.DET=100*sum(diag_hist(diag_hist>1))/sum(diag_hist);
    RESULTS.MeanL=mean(diag_hist(diag_hist>1));
    RESULTS.MaxL=max(diag_hist);
```

```
[count,bin]=hist(diag_hist(diag_hist>1),min(diag_hist(diag_hist>1)):max(diag_hist));
    total=sum(count);
    p=count./total;
    del=find(count==0); p(del)=[];
    RESULTS.EntrL=-sum(p.*log2(p));
    RESULTS.LAM=100*sum(vertical_hist(vertical_hist>1))/sum(vertical_hist);
    RESULTS.MeanV=mean(vertical_hist(vertical_hist>1));
    RESULTS.MaxV=max(vertical_hist);

[count,bin]=hist(vertical_hist(vertical_hist>1),min(vertical_hist(vertical_hist>1)):max(vertical_hist));
    total=sum(count);
    p=count./total;
    del=find(count==0); p(del)=[];
    RESULTS.EntrV=-sum(p.*log2(p));
    RESULTS.EntrW=RQA_WeightedEntropy( wrp );
else
    RESULTS.DET=NaN;
    RESULTS.MeanL=NaN;
    RESULTS.MaxL=NaN;
    RESULTS.EntrL=NaN;
    RESULTS.LAM=NaN;
    RESULTS.MeanV=NaN;
    RESULTS.MaxV=NaN;
    RESULTS.EntrV=NaN;
    RESULTS.EntrW=NaN;
end
```

```
RP=imrotate(1-A,90);

%% PLOT
scrsz = get(0,'ScreenSize');
f = figure('Position',[scrsz(3)/4 scrsz(4)/4 scrsz(3)/3 scrsz(4)/2]);tabgp =
uitabgroup(f);
binary = uitab(tabgp,'Title','Binary');
heatmap = uitab(tabgp,'Title','Heatmap');
% Binary Plot (Tab 1)
a1 = axes('Parent',binary,'Position', [0 0 1 1], 'Visible', 'off');
ax(1) = axes('Parent',binary,'Position',[.375 .35 .58 .6], 'FontSize', 8);
imagesc(ax(1),RP); colormap(gray);
title(['DIM = ', num2str(DIM), '; EMB = ',num2str(EMB), '; DEL = ',
num2str(DEL), '; RAD = ', num2str(radius), '; NORM = ',num2str(NORM), ';
ZSCORE = ',num2str(ZSCORE)],'FontSize',8)
xlabel('X(i)','Interpreter','none', 'FontSize', 10);
ylabel('Y(j)','Interpreter','none', 'FontSize', 10);
set(gca,'XTick',[ ]);
set(gca,'YTick',[ ]);
switch upper(TYPE)
    case {'RQA','MDRQA','MD','MULTI'}
        ax(2) = axes('Parent',binary,'Position',[.375 .1 .58 .15],
'FontSize', 8);
        plot(1:length(DATA(:,1)), DATA(:,1), 'k-');
        xlim([1 length(DATA(:,1))]);
        ax(3) = axes('Parent',binary,'Position',[.09 .35 .15 .6], 'FontSize',
8);
        plot(DATA(:,1), 1:length(DATA(:,1)), 'k-');
```

```
        ylim([1 length(DATA(:,1))]);
    case {'CRQA','CROSS'}
        ax(2) = axes('Parent',binary,'Position',[.375 .1 .58 .15],
'FontSize', 8);
        plot(1:length(DATA(:,1)), DATA(:,1), 'k-');
        xlim([1 length(DATA(:,1))]);
        ax(3) = axes('Parent',binary,'Position',[.09 .35 .15 .6], 'FontSize',
8);
        plot(DATA(:,2), 1:length(DATA(:,2)), 'k-');
        ylim([1 length(DATA(:,1))]);
    case {'JRQA','JOINT'}
        for i = 1:c
            ax(2) = axes('Parent',binary,'Position',[.375 .1 .58 .15],
'FontSize', 8);
            plot(1:length(DATA(:,1)), DATA(:,i),'k-');
            xlim([1 length(DATA(:,1))]);
            ax(3) = axes('Parent',binary,'Position',[.09 .35 .15 .6],
'FontSize', 8);
            plot(DATA(:,1), 1:length(DATA(:,i)),'k-');
            ylim([1 length(DATA(:,1))]);
        end
end
set(gcf, 'CurrentAxes', a1);
str(1) = {['%REC = ', sprintf('%.2f',RESULTS.REC)]};
text(.1, 0.27, str, 'FontSize', 8, 'Color', 'k');
str(1) = {['%DET = ', sprintf('%.2f',RESULTS.DET)]};
text(.1, .24, str, 'FontSize', 8, 'Color', 'k');
str(1) = {['MaxL = ', sprintf('%.0f',RESULTS.MaxL)]};
text(.1, .21, str, 'FontSize', 8, 'Color', 'k');
```

```
str(1) = {['MeanL = ', sprintf('%.2f',RESULTS.MeanL)]};
text(.1, .18, str, 'FontSize', 8, 'Color', 'k');
str(1) = {['EntrL = ', sprintf('%.2f',RESULTS.EntrL)]};
text(.1, .15, str, 'FontSize', 8, 'Color', 'k');
str(1) = {['%LAM = ', sprintf('%.2f',RESULTS.LAM)]};
text(.1, .12, str, 'FontSize', 8, 'Color', 'k');
str(1) = {['MaxV = ', sprintf('%.0f',RESULTS.MaxV)]};
text(.1, .09, str, 'FontSize', 8, 'Color', 'k');
str(1) = {['MeanV = ', sprintf('%.2f',RESULTS.MeanV)]};
text(.1, .06, str, 'FontSize', 8, 'Color', 'k');
str(1) = {['EntrV = ', sprintf('%.2f',RESULTS.EntrV)]};
text(.1, .03, str, 'FontSize', 8, 'Color', 'k');

% Heatmap Plot (Tab 2)
a2 = axes('Parent',heatmap,'Position', [0 0 1 1], 'Visible', 'off');
ax(4) = axes('Parent',heatmap,'Position',[.375 .35 .58 .6], 'FontSize', 8);
imagesc(ax(4),imrotate(-1*wrp,90));
title(['DIM = ', num2str(DIM), '; EMB = ',num2str(EMB), '; DEL = ',
num2str(DEL), '; RAD = ', num2str(radius), '; NORM = ',num2str(NORM), ';
ZSCORE = ',num2str(ZSCORE)],'FontSize',8)
xlabel('X(i)','Interpreter','none', 'FontSize', 10);
ylabel('Y(j)','Interpreter','none', 'FontSize', 10);
set(gca,'XTick',[ ]);
set(gca,'YTick',[ ]);
switch upper(TYPE)
    case {'RQA','MDRQA','MD','MULTI'}
        ax(5) = axes('Parent',heatmap,'Position',[.375 .1 .58 .15],
'FontSize', 8);
```

```
        plot(1:length(DATA(:,1)), DATA(:,1), 'k-');
        xlim([1 length(DATA(:,1))]);
        ax(6) = axes('Parent',heatmap,'Position',[.09 .35 .15 .6],
'FontSize', 8);
        plot(DATA(:,1), 1:length(DATA(:,1)), 'k-');
        ylim([1 length(DATA(:,1))]);
    case {'CRQA','CROSS'}
        ax(5) = axes('Parent',heatmap,'Position',[.375 .1 .58 .15],
'FontSize', 8);
        plot(1:length(DATA(:,1)), DATA(:,1), 'k-');
        xlim([1 length(DATA(:,1))]);
        ax(6) = axes('Parent',heatmap,'Position',[.09 .35 .15 .6],
'FontSize', 8);
        plot(DATA(:,2), 1:length(DATA(:,2)), 'k-');
        ylim([1 length(DATA(:,1))]);

    case {'JRQA','JOINT'}
        for i = 1:c
            ax(5) = axes('Parent',heatmap,'Position',[.375 .1 .58 .15],
'FontSize', 8);
            plot(1:length(DATA(:,1)), DATA(:,i),'k-');
            xlim([1 length(DATA(:,1))]);
            ax(6) = axes('Parent',heatmap,'Position',[.09 .35 .15 .6],
'FontSize', 8);
            plot(DATA(:,1), 1:length(DATA(:,i)),'k-');
            ylim([1 length(DATA(:,1))]);
        end
end
```

```
set(gcf, 'CurrentAxes', a2);
str(1) = {['EntrW = ', sprintf('%.2f',RESULTS.EntrW)]};
text(.1, 0.27, str, 'FontSize', 8, 'Color', 'k');
linkaxes(ax([1,4]),'xy');
linkaxes(ax([1,2,4,5]),'x');
linkaxes(ax([1,3,4,6]),'y');

%% Function for setting radius to achieve a certain recurrence
    function [perRec, diag_hist, vertical_hist, radiusFinal,A] =
setRadius(radius)
        % Find the radius to provide target % recurrence
        [perRec, ~, ~, ~] = recurrenceMethod(a,radius);
        while perRec == 0 || perRec > 2.5
            % if radius is too small
            display('Minimum radius has been adjusted...');
            if perRec == 0
                radius = radius*2;
            elseif perRec > SETVALUE
                radius = radius / 1.5;
            end
            [perRec, ~, ~, ~] = recurrenceMethod(a,radius);
        end

        [perRec, ~, ~, ~] = recurrenceMethod(a,radiusEnd);
        while perRec < SETVALUE
            % if radiusEnd is too large
            display('Maximum radius has been increased...');
            radiusEnd = radiusEnd*2;
```

```
            [perRec, ~, ~, ~] = recurrenceMethod(a,radiusEnd);
        end

        % Search for radius with target % recurrence
        wb = waitbar(0,['Finding radius to give %REC = ',num2str(SETVALUE), ' Please wait...']);    % create wait bar to display progress
        lv = radius;    % set low value
        hv = radiusEnd; % set high value
        target = SETVALUE;  % designate what percent recurrence is wanted
        iter = 20; % Number of iterations to find radius
        for  i1 = 1:iter
            mid(i1) = (lv(i1)+hv(i1))/2;   % find midpoint between hv and lv
            rad(i1) = mid(i1);    % new radius for this iteration
            %Compute recurrence matrix
            [perRec, diag_hist, vertical_hist,A] = recurrenceMethod(a, rad(i1));

            perRecIter(i1) = perRec;  % set percent recurrence

            if perRecIter(i1) < target
                % if percent recurrence is below target percent recurrence
                hv(i1+1) = hv(i1);
                lv(i1+1) = mid(i1);
            else
                % if percent recurrence is above or equal to target percent recurrence
                lv(i1+1) = lv(i1);
                hv(i1+1) = mid(i1);
```

```
                end
                waitbar(i1/iter,wb); % update wait bar
            end

            close(wb)
            perRecFinal = perRecIter(end);  % set final percent recurrence
            radiusFinal = rad(end);       % set radius for final percent recurrence
            disp(['% recurrence = ',num2str(perRecFinal),', radius = ',num2str((radiusFinal))])
        end

        function [perRec, diag_hist, vertical_hist, A] = recurrenceMethod(A, radius)
            if ~iscell(A)
                A = {A};
            end
            for i2 = 1:length(A)
                A{i2} = A{i2}+radius;
                A{i2}(A{i2} >= 0) = 1;
                A{i2}(A{i2} < 0) = 0;
            end

            if length(A) > 1
                for i3 = 1:length(A)-1
                    A{i3+1} = A{i3}.*A{i3+1};
                end
                A = A{i3+1};
```

```
        else
            A = A{1};
        end

        diag_hist = [];
        vertical_hist = [];
        for i4 = -(length(DATA)-1):length(DATA)-1 % caluculate diagonal line
distribution
            C=diag(A,i4);
            % bwlabel is taking each diagonal line and looking for the 1's,
it will
            % return increasing numbers for each new instance of 1's, for
example
            % the input vector [0 1 1 0 1 0 1 1 0 0 1 1 1] will return
            %                  [0 1 1 0 2 0 3 3 0 0 4 4 4]
            d=bwlabel(C,8);
            % tabulate counts the instances of each integer, therefore the
line
            % lengths
            d=tabulate(d);
            if d(1,1)==0
                d=d(2:end,2);
            else
                d=d(2);
            end
            % diag_hist is creating one long array of all of the line lengths
for
            % all of the diagonals
```

```
            diag_hist(length(diag_hist)+1:length(diag_hist)+length(d))=d;
        end

        % This removes the line of identity in RQA, jRQA, and mdRQA
        if ~contains(upper(TYPE),{'CROSS','CRQA'})
            diag_hist=diag_hist(diag_hist<max(diag_hist));
            if isempty(diag_hist)
                diag_hist=0;
            end
        end

        for i5=1:length(DATA) % calculate vertical line distribution
            C=(A(:,i5));
            v=bwlabel(C,8);
            v=tabulate(v);
            if v(1,1)==0
                v=v(2:end,2);
            else
                v=v(2);
            end

vertical_hist(length(vertical_hist)+1:length(vertical_hist)+length(v))=v;
        end

        % Calculate percent recurrence
        if ~contains(upper(TYPE),{'CROSS','CRQA'})
            perRec = 100*(sum(sum(A))-length(A))/(length(A)^2-length(A));
        else
```

```
            perRec = 100*(sum(sum(A)))/(length(A)^2);
        end
    end
%% Calculate entropy of weighted recurrence plot
    function [ Swrp ] = RQA_WeightedEntropy( WRP )
        N = length(WRP);
        for j = 1:N
            si(j) = sum(WRP(:,j));
        end
        mi = min(si);
        ma = max(si);
        m = (ma - mi)/49;
        I = 1;
        S = sum(si);
        for s = mi:m:ma
            P = sum( si(si >= s & si < (s+m)) );
            p1( I ) = P / S;
            I = I+1;
        end
        for I = 1:length(p1)
            pp(I) = (p1(I)*log(p1(I)));
        end
        pp(isnan(pp)) = 0;
        Swrp = -1*(sum(pp));
    end
end
```

Matlab script used to calculate number of right heel strikes in each breath

```
function [ DRPss,freq ] = drp( B, EI, W, saveFigs, outputFilename )
%   DRP is a function to give frequency and phasic relationships between
%   two time series.
%
%   Last updated by Will Denton: 2016-04-12
%   Contact information: 21denton@gmail.com
%
%   [ DRPss,freq ] = drp( B, W, saveFigs, outputFilename ) reads vectors
%   B and W, two time series. DRP figures are output if saveFigs = 'save'
%   using the name input as outputFilename. DRPss outputs t, n, and T
%   values as well as the DRP value calculated from the equation t/T*360°,
%   where t is the distance from the peak of the low frequency oscillator to
%   a previous peak of the higher frequency oscillator within the same
%   cycle and T is the distance between the same peak of the higher
%   frequency oscillator and the following peak of the higher frequency
%   oscillator. The output variable freq gives the percentage of
%   frequencies spent at integer and half integer ratios.
%
%   Two consecutive cycles (of the lower frequency oscillator) must be at
%   the same frequency to be counted as an integer ratio. To be counted as
%   a half integer ratio, the frequency must bounce back and forth between
%   two frequencies for at least three cycles (ex. 2 1 2 ==> 1.5 1.5 1.5).
%   If the frequency is only seen in one consecutive cycle, it is
%   considered as non-coupled and given a value of 0.

    [~,HS] = findpeaks(W);
```

```
%%
F1 = figure('units','normalized','outerposition',[0 0 1 1]);
tgroup = uitabgroup('Parent', F1);
tab1 = uitab('Parent', tgroup, 'Title', 'DRP');
tab2 = uitab('Parent', tgroup, 'Title', 'Return Map');
tab3 = uitab('Parent', tgroup, 'Title', 'Rose Plot');

%% Tab 1
a1 = axes('parent', tab1);
subplot(3,2,1); plot(B,'k'); hold on;
for i = 1:length(HS)
    plot([HS(i),HS(i)],[min(B),max(B)],'k');
end
set(gca,'XLim',[0 length(B)]);
set(gca,'YLim',[mean(B)-0.5*std(B) max(B)]);
scatter(EI,B(EI),'k','filled');

%% DRP calculation:
%% Compute tiR(p)
clc; i = 2; back = 0;
for i1 = 1:length(HS)
    if HS(i1) < EI(1)
        display('Discrete point discarded ...');
        back = back+1;
    elseif HS(i1) < EI(i)
        tiRp(i1-back) = EI(i) - HS(i1);
    else
```

```
            if i < length(EI)
                i = i+1;
                tiRp(i1-back) = EI(i) - HS(i1);
            end
        end
    end
    HS = HS(back+1:end);
    HS = HS(HS <= EI(end));

    %% Compute Ti
    Ti = diff(HS);

    %% Find n's
    for i = 1:length(EI) - 1
         holder = 0;
             for f = 1:length(HS)-1
                 if HS(f) >= EI(i) && HS(f) < EI(i+1)
                     holder = holder + 1;
                 end
             end
         n(i,1) = holder-1;
    end

    %% Discrete relative phase
    i = 1;
    DRPss(1,:) = {'t','n','T','DRP'};
    for i1 = 1:length(Ti)
        DRP(i1,1) = tiRp(i1)/Ti(i1)*360;
```

```
        DRPss{i+1,1} = tiRp(i1);
        DRPss{i+1,3} = Ti(i1);
        DRPss{i+1,4} = DRP(i);
        i = i+1;
    end

    i = 1;
    for k = 1:length(n)
        for i1 = 0:n(k)
            DRPss{i+1,2} = n(k) - i1;
            i = i+1;
        end
    end

    subplot(3,2,3:4); plot(DRP,'k-o'); hold on;
    maxDRP = round(max(DRP)/360)+1;
    for i = 1:maxDRP
        plot([0,length(DRP)],[360*(i-1),360*(i-1)],'k');
        for i1 = 1:length(DRP)
            if DRP(i1) > 360*(i-1) && DRP(i1) <= 360*i
                count(i1,1) = i;
            else
            end
        end
    end
    set(gca,'XLim',[1 length(DRP)]); set(gca,'YTick',0:360:360*DRP);
set(gca,'YLim',[0 360*maxDRP]);
```

```
    %% Find half integers
    DRPfirst(1,1) = DRPss{2,4};
    i1 = 2;
    for i = 3:length(DRPss)-1
        if DRPss{i,2} == 0
            DRPfirst(i1,1) = DRPss{i+1,4};
            DRProse(i1,1) = DRPss{i,4};
            i1 = i1+1;
        else
        end
    end

    %% Get percentage of coupling frequencies
    for i = 1:length(DRPfirst)
        for i1 = 1:maxDRP
            if DRPfirst(i) > (i1-1) * 360 && DRPfirst(i) <= i1*360
                freqFromDRP(i) = i1;
            else
            end
        end
    end

    if freqFromDRP(1) == freqFromDRP(3) && freqFromDRP(2) == freqFromDRP(4)
&& abs( freqFromDRP(1)-freqFromDRP(2) ) == 1
        DRPfirstHalfInt(1) = ( freqFromDRP(1) + freqFromDRP(2) ) / 2;
    else
        DRPfirstHalfInt(1) = freqFromDRP(1);
    end
```

```
    last=length(freqFromDRP); counter = 0;
    for i = 2:last-1
        if freqFromDRP(i-1) == freqFromDRP(i+1) && abs(freqFromDRP(i)-freqFromDRP(i+1)) == 1
            DRPfirstHalfInt(i) = ( freqFromDRP(i)+freqFromDRP(i+1) ) / 2;
            counter = counter+1;
        else
            if mod(counter,2) == 1 && counter<i && counter > 1
                DRPfirstHalfInt(i-counter-1) = DRPfirstHalfInt(i-counter);
            else
            end
            DRPfirstHalfInt(i) = freqFromDRP(i);
            counter = 0;
        end
    end
    DRPfirstHalfInt(last) = freqFromDRP(last);

    %First
    if DRPfirstHalfInt(1) == DRPfirstHalfInt(2)
        DRPwithNC(1) = DRPfirstHalfInt(1);
    else
        DRPwithNC(1) = 0; %NC
    end
    %Middle
    for i = 2:length(DRPfirstHalfInt)-1;
        if DRPfirstHalfInt(i) == DRPfirstHalfInt(i+1) || DRPfirstHalfInt(i) == DRPfirstHalfInt(i-1)
```

```matlab
                DRPwithNC(i) = DRPfirstHalfInt(i);
            else
                DRPwithNC(i) = 0; %NC
            end
        end
        %End
        last = length(DRPfirstHalfInt);
        if DRPfirstHalfInt(last) == DRPfirstHalfInt(last-1)
            DRPwithNC(last) = DRPfirstHalfInt(last);
        else
            DRPwithNC(last) = 0; %NC
        end

        subplot(3,2,5:6); plot(DRPfirst,'k-o'); hold on;
        maxDRP = round(max(DRPfirst)/360)+1;
        for i = 1:maxDRP
            plot([0,length(DRPfirst)],[360*i,360*i],'k');
            for i1 = 1:length(DRPfirst)
                if DRPfirst(i1) > 360*(i-1) && DRPfirst(i1) <= 360*i
                    count(i1,1) = i;
                    text(i1,DRPfirst(i1),['
',num2str(DRPwithNC(i1))],'VerticalAlignment','bottom');
                else
                end
            end
        end
        set(gca,'XLim',[1 length(DRPfirst)]); set(gca,'YTick',0:360:360*maxDRP);
set(gca,'YLim',[0 360*maxDRP]);
```

```
%%
maxn = max(DRPwithNC);
freq(1,:) = {'Ratio','Occurances','PercentageofTotal'};
freq{2,1} = 0; %NC
freq{2,2} = length(DRPwithNC(DRPwithNC==0));
freq{2,3} = freq{2,2}/length(DRPwithNC)*100;
for i = [1:0.5:maxn]
    freq{i*2+1,1} = i;
    freq{i*2+1,2} = length(DRPwithNC(DRPwithNC==i));
    freq{i*2+1,3} = freq{i*2+1,2}/length(DRPwithNC)*100;
end

%%
[B,~] = size(freq);
subplot(3,2,2);
for i = 2:B
    bar(freq{i,1},freq{i,3},0.5,'k'); hold on;
    if freq{i,3} > 0
        text(freq{i,1}-0.025*B,-1,sprintf('%2.2f%%',(freq{i,3})),'VerticalAlignment','bottom','Color','w','FontSize',12,'FontWeight','bold');
    end
end
holder = freq{i,1}+0.5;

set(gca,'XLim',[-0.5 maxn+1]);
set(gca,'XTick',[(0:0.5:maxn+0.5)]);
```

```
set(gca,'XTickLabel',{'NC',(0.5:0.5:maxn),'PC'});
set(gca,'YTick',[0:10:100]);
set(gca,'YLim',[0 100]);

%%
[a,~] = size(freq);
for i = 2:a
    c(i) = freq{i,3};
end
[MCFRpercent,MCFRindex] = max(c(2:length(c)));
MCFR = freq{MCFRindex+1,1};

%% Check which frequencies are present;
clear f;
for i = 3:length(freq)
    if freq{i,3} ~= 0 && freq{i,3} <= 100
        if mod(freq{i,1},1) ~= 0
            mult = 2;
        else
            mult = 1;
        end
        if exist('f')
            f = [f,freq{i,1}*mult];
        else
            f = freq{i,1}*mult;
        end
    end
end
```

```
    f = sort(f);

    [~,location] = max([freq{3:length(freq),2}]);
    location = location+1;
    maxLag = location+1;
    if mod(location,2) == 0
      location = location/2;
    end
    maxLag4PC = round(freq{maxLag,1});

    %% Return maps
    n=1;
    for lag = location
        original = DRP(1:length(DRP)-lag);
        lagged = DRP(1+lag:length(DRP));

        %% For each 360-multiple
        for k = 1:maxLag4PC
            original_dn = original(original <= 360*k & lagged <=360*k);
            lagged_dn = lagged(original <= 360*k & lagged <=360*k);
            dn = ( (original_dn - lagged_dn) * cosd(45) );

            for i = 1:length(dn)
                if abs(dn(i)) <= 40 %*cosd(45)
                    wdn(i) = 1 - ( abs(dn(i)) / (40)); %*cosd(45)) );
                else
                    wdn(i) = 0;
                end
```

```
            end
            PC = sum(wdn) / length(wdn) * 100;
            LagPC(k,:) = [lag,PC];
            n = n+1;
        end
    end

    [~,lag4maxPC] = max(LagPC(:,2));
    index = LagPC(lag4maxPC,1);
    [r,~] = size(freq);
    freq{r+1,1} = '---';
    freq{r+1,2} = '---';
    freq{r+1,3} = '---';
    freq{r+2,1} = 'PC';
    freq{r+2,2} = index;
    freq{r+2,3} = max(LagPC(:,2));
    subplot(3,2,2); hold on; bar(holder,LagPC(lag4maxPC,2),0.5,'k');
    text(holder-0.025*B,-
1,sprintf('%2.2f%%',LagPC(lag4maxPC,2)),'VerticalAlignment','bottom','Color',
'w','FontSize',12,'FontWeight','bold');
    original = DRP(1:length(DRP)-index);
    lagged = DRP(1+index:length(DRP));

    %% Tab 2
    a2 = axes('parent', tab2);
    scatter(original,lagged,'k'); hold on;
    plot([0 max(DRP)],[0 max(DRP)],'k'); plot([0+40 max(DRP)+40],[0
max(DRP)],'r--'); plot([0-40 max(DRP)-40],[0 max(DRP)],'r--');
```

```
    title(['Lag = ',num2str(index)]); set(gca,'XLim',[0,max(DRP)]);
set(gca,'YLim',[0,max(DRP)]); set(gca,'XTick',0:360:max(DRP));
set(gca,'YTick',0:360:max(DRP)); grid on;
    for i = 1:round(MCFR)
        annotation(tab2,'textbox',[0.1025+(0.775)/(max(DRP))*(180+360*(i-1))
, 0.075 , 0.0325 ,
0.02],'String',num2str(LagPC(i,2)),'FontSize',20,'EdgeColor','none');
    end

    %% Tab 3
    a3 = axes('parent', tab3); rose(DRProse/180*pi,20);
    [r,c] = size(freq);
    fprintf('\r');
    for i = 1:r
        for i1 = 1:c
            if i == 1
                %Do nothing
            elseif i == r-1
                fprintf('%s\t\t',freq{i,i1});
            elseif i == r

            else
                fprintf('%2.1f\t\t',freq{i,i1});
            end
        end
        if i == 1
            fprintf('%s\t\t%s\t%s',freq{i,:});
        elseif i == r
```

```
        fprintf('%s\t\t%2.2f\t\t%2.2f',freq{i,:});
    end
    fprintf('\r');
end
fprintf('\r');

filename = outputFilename;
if iscell(filename)
    filename = filename{1};
end

if  saveFigs == 1
    savefig(F1,[filename '_DRP.fig']);
    savefig(f2,[[newSub_dir filename '_' ] 'returnMap.fig'])
    save([filename '_DRP.mat'],'DRPss')
    save([filename '_freq.mat'],'freq')
else
    display('Figures not saved ...');
end

match = 0;
if exist('DRPresults.mat') ~= 0
    load('DRPresults.mat');
    [~,l] = size(DRPresults);
    for i = 1:l
        if strfind(DRPresults(i).name, filename) == 1
            l = i;
            match = 1;
```

```
            else
                if i == l && match == 0
                    l = l+1;
                end
            end
        end
    else
        l = 1;
    end
    DRPresults(l).name = filename;
    DRPresults(l).MCFR = MCFR;
    DRPresults(l).MCFRpercent = MCFRpercent;
    DRPresults(l).PC = LagPC(:,2);
    DRPresults(l).PClag = LagPC(lag4maxPC,1);
    DRPresults(l).freq = freq;
    save('DRPresults','DRPresults');
end
```
